# p300 exerts an epigenetic role in chronic neuropathic pain through its acetyltransferase activity in rats following chronic constriction injury (CCI)

**DOI:** 10.1186/1744-8069-8-84

**Published:** 2012-11-23

**Authors:** Xiao-Yan Zhu, Chang-Sheng Huang, Qian Li, Rui-min Chang, Zong-bing Song, Wang-yuan Zou, Qu-Lian Guo

**Affiliations:** 1 Department of Anesthesiology, Xiangya Hospital of Central South University, 87 Xiangya Road, Changsha City, Hunan, 410008, China; 2 Liver Cancer Laboratory, Xiangya Hospital of Central South University, 87 Xiangya Road, Changsha City, Hunan, 410008, China

**Keywords:** Neuropathic pain, p300, COX-2, Acetyltransferase activity, CCI

## Abstract

**Background:**

Neuropathic pain is detrimental to human health; however, its pathogenesis still remains largely unknown. Overexpression of pain-associated genes and increased nociceptive somato-sensitivity are well observed in neuropathic pain. The importance of epigenetic mechanisms in regulating the expression of pro- or anti-nociceptive genes has been revealed by studies recently, and we hypothesize that the transcriptional coactivator and the histone acetyltransferase E1A binding protein p300 (p300), as a part of the epigenetic mechanisms of gene regulation, may be involved in the pathogenesis of neuropathic pain induced by chronic constriction injury (CCI). To test this hypothesis, two different approaches were used in this study: (I) down-regulating p300 with specific small hairpin RNA (shRNA) and (II) chemical inhibition of p300 acetyltransferase activity by a small molecule inhibitor, C646.

**Results:**

Using the CCI rat model, we found that the p300 expression was increased in the lumbar spinal cord on day 14 after CCI. The treatment with intrathecal p300 shRNA reversed CCI-induced mechanical allodynia and thermal hyperalgesia, and suppressed the expression of cyclooxygenase-2 (COX-2), a neuropathic pain-associated factor. Furthermore, C646, an inhibitor of p300 acetyltransferase, also attenuated mechanical allodynia and thermal hyperalgesia, accompanied by a suppressed COX-2 expression, in the spinal cord.

**Conclusions:**

The results suggest that, through its acetyltransferase activity in the spinal cord after CCI, p300 epigenetically plays an important role in neuropathic pain. Inhibiting p300, using interfering RNA or C646, may be a promising approach to the development of new neuropathic pain therapies.

## Background

Neuropathic pain is a direct consequence of a lesion or disease affecting the somatosensory system at the peripheral or central level [1,2] which is characterized by spontaneous pain, hyperalgesia, allodynia, and paresthesia. The underlying mechanisms of neuropathic pain are still poorly understood, and, thus, its treatment remains a challenge. Accumulative evidence indicates that the expression of pain-associated genes in sensory neurons greatly contributes to the development and maintenance of neuropathic pain [3,4].

In the rat spinal cord, COX-2 is expressed constitutively in locations consistent with the neuroanatomical substrates of spinal nociception [5]. It is an inducible enzyme that increases significantly following an injury, lasting for several months or even several years [6,7]. Overexpression of COX-2 in injured nerve is observed in rats following CCI, partial sciatic nerve ligation, spinal nerve ligation, and complete sciatic nerve transaction in studies [7,8]. COX-2 produces prostaglandins that contribute to the development and maintenance of spinal hyperexcitability after peripheral nerve injury [5,6,9-11]. Administration of selective COX-2 inhibitor after CCI injury can attenuate the development of hyperalgesia and/or allodynia in chronic neuropathic pain in rats [11-14], and can also inhibit the CCI-induced elevation of neuronal COX-2 expression in the spinal cord [8]. All these findings demonstrate that COX-2 pathogenetically contributes to hyperalgesia and allodynia at the spinal cord level, and that the alteration of COX-2 expression is an important feature in neuropathic pain.

Epigenetics is an emerging field in biology, which reveals that stable changes in gene expression can occur without alteration in the DNA sequence, as other (epigenetic) factors are also involved in gene expression rather than genes alone. Covalent modification of the DNA-packaging histone that regulates the expression of pro- or antinociceptive genes is one of the characterized mechanisms of epigenetics [15-17]. As a histone acetyltransferase, p300 is a major contributor to the regulation of gene expression [18]. It is expressed ubiquitously and plays an important role in a wide range of biological processes. p300 regulates the expression of various kinds of genes through bridging, scaffolding or histone acetyltransferase (HAT) activity, epigenetically contributing to illnesses such as inflammation, tumor, neurodegenerative diseases and so on [19-21]. However, little is known about the expression of p300 and its role in the CCI-induced neuropathic pain. There are studies demonstrating p300’s essential role in COX-2 transcriptional activation in the cell signaling pathway using chromatin immunoprecipitation (ChIP) analysis [22,23].

Taking these facts together, we hypothesized that p300 may be epigenetically involved in neuropathic pain through regulating the expression of neuropathic pain-associated factor COX-2 in CCI rats. In this study, we examined p300 and COX-2 expression in the spinal cord in the neuropathic pain rats after CCI. We evaluated the effects of intrathecal administration of lentiviral vectors encoding small interfering RNA specifically against the p300 gene (LV-shp300) on COX-2 expression and neuropathic pain in the CCI rats. We also used C646 to inhibit p300 acetyltransferase activity [24] to further verify p300’s role in neuropathic pain.

## Results

### Verification of the transfected LV-shp300 in the cells of spinal dorsal horn

**Figure 1:**
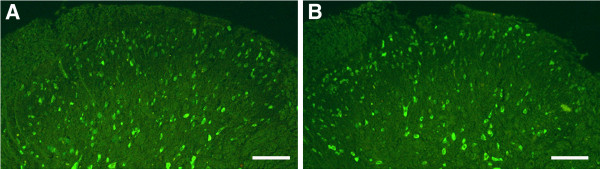
**The detection of lentiviral neurocyte transfection.** Fluorescence microscopy images of GFP expression showing transfection of lentiviral vectors into the neurons of spinal dorsal horn. GFP was detected in NC-treated rats (**A**) and LV-shp300-treated rats (**B**). Scale bars = 100um.

The lentiviral vectors that were carrying a plasmid expressing green fluorescent protein (GFP) were delivered into the subarachnoid space to transfect the cells of the spinal cord (Figure 1). The fresh sections taken from the spinal cord tissue on day 14 after CCI were observed under fluorescence microscopy. The GFP-positive cells were clearly seen in neurons in the group of lentiviral vectors encoding small interfering RNA as the negative control of p300 gene (LV-NC) and LV-shp300-treated rats.

### Changes in behavior and neuropathic pain measurements

**Figure 2:**
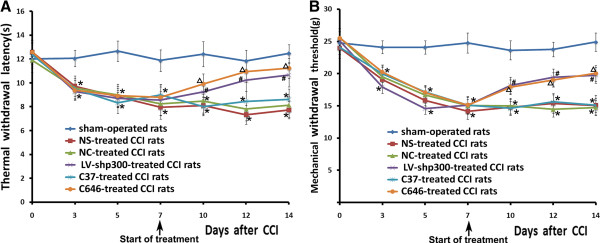
**Changes of MWT and TWL data (mean ± SEM) in all rats.A**: Changes on thermal hyperalgesia in all rats. **B**: Changes on mechanical allodynia in all rats. Comparison of TWL and MWT in rats with different treatments: *P < 0.05 vs. sham-operated group; ^#^P < 0.05 vs. NS and NC-treated groups. ^Δ^P < 0.05 vs. C37-treated group.

After CCI, the rats gradually showed the typical signs of hyperalgesia and allodynia such as toe closing, foot eversion, and paw-licking. Behaviors in sham-operated rats were not obviously changed. The changes in ipsilateral mechanical withdrawal threshold (MWT) and thermal withdrawal latency (TWL) were demonstrated in Figure 2. There was no significant difference in MWT and TWL among the groups (P > 0.05) after catheterization and before CCI surgery. MWT and TWL of CCI rats decreased on day 3 after CCI and were further reduced on day 5 compared with sham-operated rats (sham, P < 0.05), which indicated that the mechanical allodynia and thermal hyperalgesia had been developed in CCI rats. Later, in normal saline (NS)-treated group, LV-NC-treated group and C37 (inactive analog of C646)-treated group, MWT and TWL went much lower on day 7, and this persisted at the end of the study (P < 0.05). In contrast, MWT and TWL were constantly recovering from day 10 to the end of the study in LV-shp300 and C646-treated rats (P < 0.05). The inhibition of p300 with specific shRNA or C646 attenuated the pain during the steady-state of neuropathic pain in our study, suggesting a potential functional association between p300 activity and neuropathic pain during CCI.

### Changes in p300 expression

**Figure 3:**
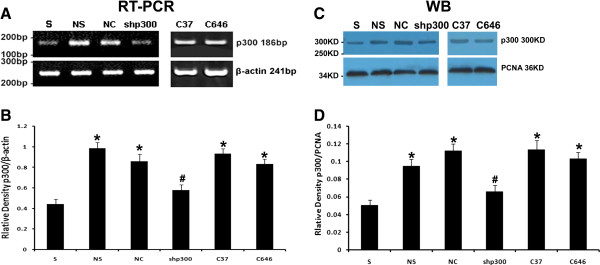
**The effects of LV-shp300 and C646 on p300 expression in the lumbar spinal cord in rats.A**: Expression of mRNAs for p300 in the lumbar spinal cord in all rats. The β-actin fragment was used as an internal control. **B**: The mean (±SEM) of integral optical density (IOD) of P300/β-actin in rats on mRNA level, n = 5. **C**: Expression of protein p300 in the lumbar spinal cord in all rats. **D**: The mean (±SEM) of IOD of P300/PCNA in all rats on protein level, n = 5. S: sham-operated rats; NS: NS-treated CCI rats; NC: LV-NC-treated CCI rats; shp300: LV-shp300-treated CCI rats. C37: C37-treated CCI rats; C646:C646-treated CCI rats. *P < 0.05 vs. sham-operated group; ^#^P < 0.05 vs. NS and NC-treated groups.

To evaluate the inhibitory effect of LV-shp300 on p300 mRNA expression in the spinal cord, reverse transcription-polymerase chain reaction (RT-PCR) was performed at the end of the study. As shown in Figure 3A, B, while sham-operated rats showed the lowest p300 mRNA expression level, NS and NC-treated rats had the highest p300 mRNA expression (P < 0.05 vs. sham control). The p300 expression was significantly decreased in LV-shp300-treated rats compared with those in NS and NC treated rats (P < 0.05), indicating that the intrathecal LV-shp300, reversed the upregulated p300 expression after CCI. Noteworthily, C646, the high specificity inhibitor of p300 acetyltransferase activity, had little suppressive effect on p300 mRNA expressions, which was similar to its inactive analog, C37 (P > 0.05).

Similarly, Western blot analysis showed that the p300 protein levels paralleled the mRNA levels (Figure 3C, D). These findings also indicated that the inhibitory effects of a single injection of lentivirus carrying p300 shRNA were persistent for at least 7 days on the spinal cord level, which is consistent with Song’s report [25]. And C646 had no role in p300 protein expression quantitatively.

**Figure 4:**
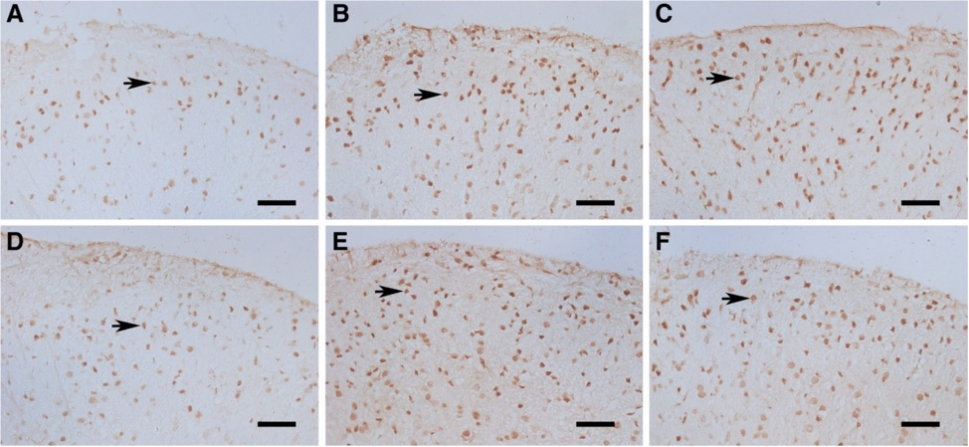
**Representative images of p300 immunoreactivity in rats’ spinal dorsal horn among different groups.** p300 immunoreactivity was mainly located in the nucleus but not in the cytoplasm. Representative p300-positive cells were pointed by arrows (Scale bars = 50um); basal p300 expression in sham-operated rats (**A**), CCI-induced overexpression of p300 in NS-treated rats (**B**), NC-treated rats (**C**), C37-treated rats (**E**) and C646-treated rats (**F**), p300 shRNA moderately decreased the p300 expression in LV-shp300-treated CCI rats (**D**).

The findings in immunostaining of p300 were also consistent with RT- PCR and Western blot results. As shown in Figure 4, p300 immunoreactivity was seen mainly in the nucleus in the superficial laminas of spinal dorsal horn in rats. Increased immunoreactivity throughout the ipsilateral dorsal horn in vehicle (NS, NC and C37)-treated CCI rats were evidenced in Figure 4B, C and E, compared with that in sham-operated group (Figure 4A). The increased p300 immunoreactivity was particularly concentrated at layers I-III, which is one of the major areas for nociceptive signal processing. p300 immunoreactivity was moderately decreased in LV-shp300-treated CCI rats (Figure 4D). Images of the C646-treated group were similar to those in vehicle-treated CCI group (Figure 4F). The overexpression of p300 in the lumbar spinal cord is similar to Matsumoto’s report [26].

### LV-shp300 and C646 injection attenuated CCI-induced p300 protein recruitment to COX-2 promoter region

**Figure 5:**
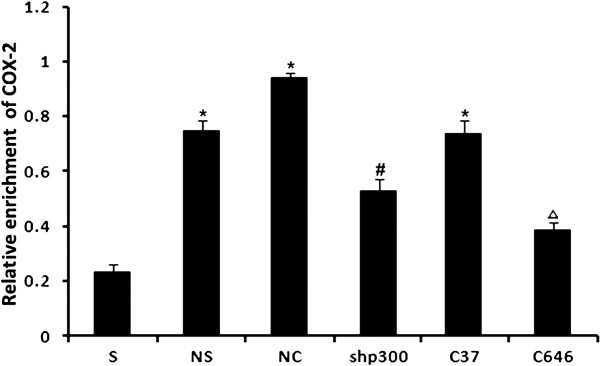
**ChIP assays in shRNA treated and p300 acetyltransferase inhibitor treated rats.** Reactions were performed with normal mouse IgG or anti-p300 antibodies. Real time PCR was used to quantify the enrichment of p300 promoter region of the cox-2 gene. Comparison between rats with different treatments: *P < 0.05 vs. sham-operated group; ^#^P < 0.05 vs. NS and NC-treated groups. ^Δ^P < 0.05 vs. C37-treated group. S: sham-operated rats; NS: NS-treated CCI rats; NC: LV-NC-treated CCI rats; shp300: LV-shp300-treated CCI rats. C37: C37-treated CCI rats; C646:C646-treated CCI rats.

The effect of CCI on p300 (protein) binding to COX-2 promoter was evaluated by ChIP assay. p300 was immunoprecipitated with specific antibodies, and the COX-2 promoter region containing the essential binding sites for promoter activation was detected by real time PCR. In order to quantify the relative amount of p300 associated with COX-2 promoter, total input DNA and the immunoprecipitated DNA were used as templates for PCR with primers for the rat COX-2 gene promoter. As shown in Figure 5, p300 binding to chromatin COX-2 promoter region was increased significantly in the groups of NS, NC, and C37-treated rats compared with the group of sham-operated rats(P < 0.05). However, it was decreased significantly in the groups treated with p300 shRNA and C646 (P < 0.05). The decreases were about 56.21% in p300 shRNA group (versus NC group) and 52.24% in C646 group (versus C37 group) of that induced by CCI. These results demonstrated that both the LV-shp300 and the C646 attenuated this CCI-induced p300 binding to the COX-2 promoter region.

### LV-shp300 and C646 injection decreased CCI-induced COX-2 expression

**Figure 6:**
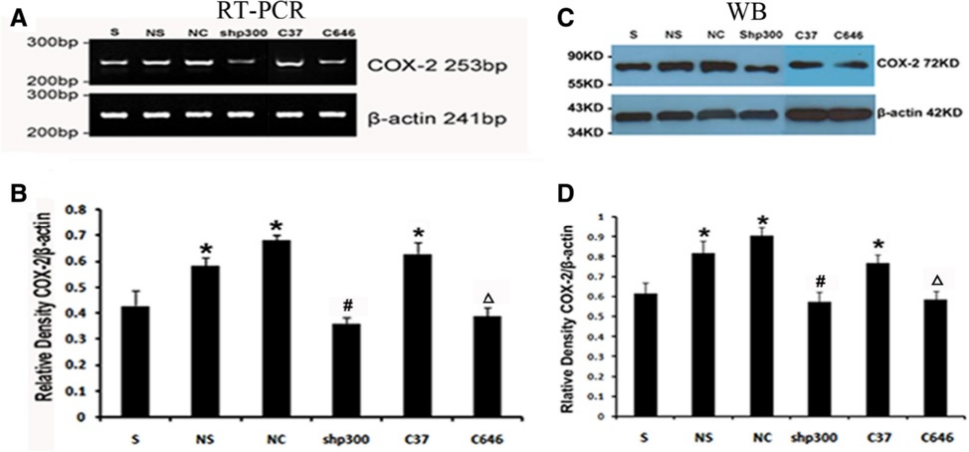
**The effects of intrathecal injections of p300 shRNA and p300 acetyltransferase inhibitor on COX-2 expression.A**: Expression of COX-2 mRNA in the lumbar spinal cord in all rats. Typical results are shown. **B**: The mean (±SEM) of IOD of COX-2/β-actin in all rats on mRNA level, n = 5; **C**: Expression of protein COX-2 in the lumbar spinal cord in all rats. Typical results are shown. **D**: The mean (±SEM) of IOD of COX-2/β-actin in all rats on protein level, n = 5. S: sham-operated rats; NS: NS-treated CCI rats; NC: LV-NC-treated CCI rats; shp300: LV-shp300-treated CCI rats. C37: C37-treated CCI rats; C646: C646-treated CCI rats. *P < 0.05 vs. sham-operated group; ^#^P < 0.05 vs. NS and NC-treated groups. ^Δ^P < 0.05 vs. C37-treated group.

RT-PCR analysis demonstrated clear expression of COX-2 and β-actin mRNA in the lumbar spinal cord among the groups. As shown in Figure 6A, B, sham-operated rats showed basic COX-2 mRNA expression level, and significant increases were seen in NS, NC and C37-treated CCI rats (P < 0.05 vs. sham control). COX-2 expression was significantly decreased in shp300 and C646 treated rats compared with the vehicle treated CCI rats (P < 0.05).

**Figure 7:**
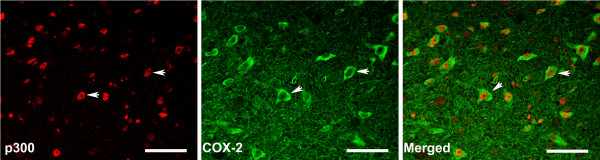
**Double immunofluorescence staining shows that p300 (red) co-localized with COX-2 (green) in the dorsal horn of the spinal cord.** Arrows indicate the representative cells expressing both p300 andCOX-2 (Scale bars = 60um).

Western blot analysis revealed that the COX-2 expression on protein level was consistent with those from RT-PCR. As shown in Figure 6C, D, the proteins of COX-2 in the lumbar spinal cord in all rats 14 days following CCI were expressed clearly. There were significant differences among the groups: COX-2 expression was increased significantly in NS-treated and NC-treated rats (P < 0.05, vs. sham control), whereas it was significantly decreased in LV-shp300-treated rats (P < 0.05). COX-2 expression in C646-treated rats also decreased (P < 0.05, vs. C37-treated group), suggesting that p300 regulates COX-2 expression probably through its acetyltransferase activity, C646 directly inhibits the acetyltransferase activity of p300, and thus, suppresses COX-2 expression. The double immunofluorescence staining of p300 and COX-2 indicated that p300 co-localized with COX-2 in the spinal dorsal horn neurons (Figure 7), serving as evidence supporting an interaction between p300 and COX-2.

## Discussion

Intrathecal application of shRNA has become a suitable approach for studies on small molecules such as drugs, receptors, or other proteins [25,27,28]. Small interference RNA (siRNA)-induced knockdown can be generated by the expression of the vector-mediated siRNA in the genome [29]. In this study, we adopted the system of lentiviral vector-mediated hairpin RNAs as it has a more efficient transfection rate in tissues in vivo compared with the system based on polyethylenimine/DNA complexes. Our results showed that LV-shp300 was successful transfected into the cells in the spinal cord (Figure 1), and it significantly silenced the p300 gene in LV-shp300-treated rats (Figure 3).

CCI induces neuropathy, hyperalgesia and allodynia normally develop on the 3^rd^ day and stabilize on the 7^th^ day after surgery [30]. In the present study, to ensure a steady-state of the pain, we began our treatments from the 7^th^ day following CCI. In the LV-shp300 and C646 treated groups, behavioral testing revealed that both the mechanical allodynia and thermal hyperexcitability gradually recovered to the baseline level, and reached their peaks on the 14^th^ day, indicating that these two p300 inhibition interferences had exerted their effects. Therefore, at the maximum time point of the functional changes, our study was mainly focused on the potential mechanism.

Functioning as an integrator for many signal transduction pathways through interaction with numerous critical transcription factors and other functional proteins, p300 is found to be highly sensitive to promoters and potential enhancers. The binding for p300 analyzed by ChIP, followed by sequencing, is positively correlated with gene expression and acetylation levels, suggesting that p300 is involved in transcriptional initiation [31]. As epigenetic processes such as histone acetylation and RNA interference have profound effects on inflammatory cytokine metabolism, steroid responsiveness, and opioid sensitivity, these processes are likely essential in the development of chronic pain [32,33]. DNA is wrapped around histone proteins, composing the chromatin; chromatin remodeling occurs through chemical modifications of histone proteins. These modifications decide how tightly or loosely the chromatin is packaged, thus controlling gene accessibility and expression [34]. p300, as a HAT, via acetylating the N-terminal tail lysines of core histones correlates strongly with active transcription of genes [35,36].

To identify the cellular and epigenetic functions relevant to p300 activities, we looked into the possibility of a molecular interaction between p300 and COX-2. COX-2, an inducible enzyme, strongly expresses in the spinal cord under proinflammatory stimulation. It is crucial in the generation of neuropathic pain [37] and in the maintenance of pain states [11]. Inflammation and nerve degeneration, seen as direct effects of nerve injury, are critical in the development of neuropathic pain states [9,38,39]. COX-2 regulates the synthesis of multiple inflammatory mediators by producing prostaglandins that are potent algetic factors in sensitizing neuronal responses. The resultant prostaglandin production augments neuronal excitability in the spinal cord (central sensitization) and in somatosensory pathways [40]. On the contrary, administering COX-2 inhibitor or inhibiting COX-2 activity decreases neuropathy-induced prostaglandin levels and reduces generated pain hypersensitivity [41].

The effect of p300 on the expression of COX-2 gene was monitored in the present study. The results revealed that the COX-2 gene expression was regulated at the chromosomal level, and the p300 recruitment to COX-2 promoter region determined the level of COX-2 gene expression in the CCI rats. These results were consistent with the previous studies indicating that p300 is essential for the regulation of COX-2 promoter activity [42-45]. Moreover, with the aid of acetylating the lysine residues in the DNA binding regions, p300 was found to regulate the expression of other pain-associated genes, such as c-Jun, in another report [46].

We also found that the binding of p300 to COX-2 was enhanced in vehicle treated CCI rats and further augmented by a p300 overexpression. The enhanced p300 binding to COX-2 was correlated with an up-regulation of COX-2 promoter activities. This enhancement in p300 activities was abrogated by p300shRNA. These results indicated p300’s importance in regulating COX-2 transcription in the neuropathic pain model. Our results also showed that the p300-mediated COX-2 transcriptional activation was dependent on its own HAT. The inhibition of the HAT by C646 resulted in an approximate reduction of 52.24% in COX-2 promoter activity. p300 HAT contributes to the accessibility of COX-2 promoter regulatory elements for transactivators to bind. Therefore, it is reasonable to conclude that the overexpression of p300 in CCI rats actually enhances the downstream transcription of pain-associated gene (such as COX-2), and the upregulation of p300 expression in the spinal cord may be a critical process in neuropathic pain induced by CCI. In summary, the importance of epigenetic mechanism for the development of chronic neuropathic pain is clearly evidenced by the changes of p300 expression during the course of neuropathy after CCI and through inhibition of p300 expression. This is additionally highlighted by the effect of p300 on COX-2 expression in this study even though detailed information on p300’s role in chronic neuropathic pain has yet to be revealed.

C646 is capable of specifically inhibiting the HAT domain of p300. It can produce 86% inhibition of p300 in vitro specifically [24] and can work well in the central nervous system in vivo [47]. p300 regulates the downstream DNA expression, not only via binding to transcription factors, but also through its acetyltransferase activity [48]. Inhibition of the histone acetyltransferase renders nucleosomal DNA less accessible to the transcriptional machinery, thereby usually favoring transcriptional silencing [49]. The blockage of p300 by C646 may lead to the inhibition of histone acetylation on the COX-2 promoter region. Inhibition of p300 with shRNA or C646 can reverse neuropathic pain and reduce COX-2 levels significantly; and their suppressive effects were similar, which is in line with the report by Santer et al. [50].

While our results show that the inhibition of histone acetylation suppresses the activity of p300 and COX-2, reducing hyperalgesia in this CCI model, there are a few studies showing that histone deacetylase (HDAC) inhibitors reduce pain hyperalgesia as a result of an increased histone acetylation [51,52]. Most of these studies focus on the relationship between HDAC and metabotropic glutamate2 (mGlu2) receptor or gamma-aminobutyric acid (GABA) synaptic function. Since individual HAT or HDAC binds on the promoter of specific genes, it may play different roles depending on the downstream target gene is pro-hyperalgesia or anti-hyperalgesia. As for p300 in the present study, it was found combined to the promoter of COX-2, one of the well-described painful genes. Moreover, even though GABA_A_ receptors have an important role in inflammatory pain mechanisms in Complete Freund’s Adjuvant (CFA) model [51], spinal GABAergic associated mechanism is found unnecessary for the development of neuropathic pain in CCI model [53]. These may explain the difference existed in the findings of others studies and ours. As the main focus of this study was to explore the potential effect of p300 and its possible epigenetic role in neuropathic pain, two different approaches were used to accomplish the goal; to down-regulate p300 with specific shRNA, and to chemically inhibit the acetyltransferase activity of p300 using C646. We did not determine the optimal dose to reverse neuropathic pain in CCI rats, nor did we compare the inhibiting efficiency with other p300 inhibitors in an equivalent dose. In addition, behavioral changes were used as the main markers for measuring the pain and treatment effects; we did not examine other signs related to antinociception or neuropathic pain. Therefore, further studies will expand this observation of neuropathic pain and p300 acetyltransferase activity into other target molecules in neuropathic pain, multi-time point and long-term model after CCI.

## Conclusions

Our results suggest that p300 may play an important role in neuropathic pain through the COX-2-associated signaling pathway in rats following CCI. The inhibition of p300 with shRNA and C646 can functionally reverse the behavior of pain and down-regulate the expression of COX-2 (a downstream gene of p300) after CCI, which suggests that p300 is epigenetically involved in neuropathic pain through its acetyltransferase activity. This provides some insights for further studies on the role of histone acetylation in neuropathic pain. Targeting p300 by HAT inhibitors may be a new therapeutic approach for neuropathic pain.

## Methods

### Animals

Male Sprague–Dawley rats (220–250 g) were purchased from the animal experiment center of Xiangya School of Medicine (Central South University, Changsha, Hunan, China). The rats were allowed to acclimate for one week after arrival. All the animals had free access to food and water in a temperature-controlled lab where the ambient temperature was maintained at 22 ± 0.5 with a light/dark cycle of 12:12 h. All animal procedures were performed in accordance with the National Institutes of Health guide for the care and use of Laboratory animals (NIH Publications No. 8023, revised 1978) and the Chinese Council’s Guide for the Care and Use of Laboratory Animals (issued in 1988). To prevent wound infection after a surgical procedure, the surgical area was dusted with streptomycin before suturing the incision in all the animals. All the protocols were approved by the Animal Care Committee of Central South University. All efforts were made to minimize animal suffering, and to reduce the numbers of animals used in this study.

### Intrathecal catheter implantation

Intrathecal catheter implantation was carried out under deep anesthesia with 10% chloral hydrate (300–350 mg/kg, i.p.). A chronic lumbar intrathecal catheter(Anilab, Ningbo, China) was implanted into the subarachnoid space in all rats as previously reported [54]. After 3 days of recovery, 2% lidocaine (10 μl) was administered intrathecally to judge whether the intrathecal catheter implantation was successful or not. Only the animals that demonstrated paralysis within 30 s and completely recovered within 30 min were used for further study [55]. The rats were housed in pairs in a 1500U rat cage that had a custom made wire fence in the middle after surgery with free access to food and water. The rats that exhibited gross motor impairment following surgery were euthanized.

### Chronic constriction injury (CCI) model

After 5 days of recovery from the implantation, all the rats showed good recovery and no signs of wound infection. The rats were randomly divided into the following groups (n = 10 each): sham-operated group, NS-treated group, LV-NC-treated group, C37-treated group, LV-shp300-treated group, and C646-treated group. Under deep anesthesia, CCI was introduced using the surgical procedure as described [56-58]. In brief, after exposing the left sciatic nerve from the surrounding tissue, four snug ligatures (4–0 chromic gut) were placed around the nerve. The interval among ligatures was about 1 mm. In the sham operation, the nerve was exposed but not ligated. To avoid variation, all the surgical procedures were performed by the same member of our team. The wounds healed gradually with no infection after the surgery.

### Construction and production of lentiviral vectors

In order to minimize off-target effects [59], a BLAST homology search based on sense and antisense sequences was systematically performed to ensure that a single mRNA sequence was targeted. The sequence for the siRNA targeting the rat p300 gene (GenBank ID:XM_576312), and for the non-silencing siRNA were as follows: shp300 (468–487 bp of p300 gene), CAGCCCAGTGAATCAACCT [60]; non-silencing siRNA, TTCTCCGAACGTGTCACGT [61]. The oligonucleotides designed according to the structure of the siRNA were p300 sense 5′- TaaCAGCCCAGTGAATCAACCTCTCGAGAGGTTGATTCACTGGGCTGttTTTTTTC-3′ and antisense 5′- TCGAGAAAAAAaaCAGCCCAGTGAATCAACCTCTCGAGAGGTTGATTCACTGGGCTGttA-3′, negative control sense 5′-TTTCTCCGAACGTGTCACGTTTCAAGAGAACGTGACACGTTCGGAGAATTTTTTC-3′ and antisense 5′-TCGAGAAAAAATTCTCCGAACGTGTCACGTTCTCTTGAAACGTGACACGTTCGGAGAAA-3′. Then the oligonucleotides containing both the siRNA and control siRNA sequences were constructed into the plasmid pGCSIL-shp300-GFP and pGCSIL-shp300 NC-GFP. The packaged plasmids were then cloned into the lentiviral vector pGCSIL-GFP (Shanghai GeneChem Co. China), and the recombinant lentiviral vectors were designated as LV-shp300 and LV-NC. To generate the lentivirus, the recombinant vector and packaged plasmids were cotransduced into 293 T cells using lipofectamine 2000 (Invitrogen, USA). Lentivirus, with the human U6 promoter carrying shp300, was generated as previously described [62]. The final titer of recombinant virus was 1 × 10^9^ TU/ml.

### Drugs and treatments

C646 (Sigma) and dimethyl sulfoxide (DMSO, Sigma) were obtained commercially. C37 (inactive analog of C646) was a gift from Dr. David Meyers (Johns Hopkins University, USA). Drug testing was delayed until the 7^th^ day after the surgery to ensure establishment of neuropathic pain prior to the initiation of drug delivery. All the drugs and treatment reagents were administrated through the lumbar intrathecal catheter. Sham-operated rats and NS-treated rats received 0.9% saline 10ul, and shRNA-treated rats received LV-NC or LV-shp300 (10 μl, diluted with nutrient medium) on day 7 after CCI. C37-treated and C646-treated rats received C37 and C646 (10 ul, diluted with 10%DMSO, one dose per day) from days 7 to 14 after CCI surgery. After the vectors or drugs were administered, the catheter was flushed with 5ul 0.9% saline.

### Neuropathic pain measurements

Ipsilateral mechanical allodynia in all rats was tested with 2390 Electronic von Frey Anesthesiometer (IITC Life Science, USA) as previously reported [63]. Thermal hyperalgesia was tested with Hargreaves Tes7370 (Ugo Basile, Comerio, Italy). The measurements were repeated three times with an interval of 5 minutes in each rat. MWT and TWL of all rats were measured before CCI, and on days 3, 5, 7, 10, 12 and 14 after CCI. All the behavioral tests were done by the same observer unaware of animal treatments.

### Sample preparation

On day 14 after CCI, the rats were killed and perfused immediately with 400 ml-500 ml of cold normal saline through the ascending aorta. Then their lumbar spinal cords were quickly removed. The tissues for ChIP, RT-PCR and western blot analysis were kept in −80°C. Tissues used for histomorphological observations were post-fixed with 4% paraformaldehyde for 8 h in 4°C. Samples from LV-NC and LV-shp300-treated rats were also made into frozen sections and observed under the fluorescent microscope.

### Chromatin immunoprecipitation (ChIP) assay

The assays were performed using the Upstate Biotechnology ChIP assay kit. The spinal cord tissue was cut into 1 mm slices and crossed-linked in 1.5% formaldehyde for 10 minutes. Then they were homogenized with phosphate-buffered saline after glycine neutralizing. The cell suspension was centrifuged at 12,000 g for 10 minutes, and SDS lysis buffer was added to the pellets. One third of the lysate was used as DNA input control. Anti-p300 antibody or non-immune rabbit IgG was used to immunoprecipitate the DNA-p300 protein complex. Immunocomplexes were collected by using protein Aagarose beads in slurry (Upstate Biotechnology, USA) after overnight antibody incubation. The precipitates were washed extensively and incubated in the elution buffer (1% SDS and 0.1 M NaHCO_3_) at 37°C for 20 minutes. Then the protein-DNA complexes were subjected to reverse cross-linking, protease digestion, and purification using a commercial kit (Qiagen, Germany).

The transcriptional start site of COX-2 is located at the 713 bp from the 5′ end of the COX-2 precursor sequence (http://www.ncbi.nlm.nih.gov/nuccore/NW_047397.1?from=12399230&to=12406343&report=genbank). The rat COX-2 DNA fragment −192/+127 was considered for the putative promoters because of containing key regulatory elements that correlates with many diseases [64,65]. In addition, the TFSearch software revealed the −49/+127 region of the COX-2 precursor sequence to be highly enriched in p300 binding sites. Therefore, the specific COX-2 primer was set at segment from −50 to +150, which contains the putative p300 binding site. Specific COX-2 promoter primers were used to amplify the rat COX-2 promoter region with following sequences: 5′primer, ACCTCTGCGATGCTCTTCCG; and 3′primer, GCTCAGGCGCTTTGCCAATA. The real time PCR was performed under standard cycling conditions (95°C for 5 min, followed by 40 cycles at 94°C for 20 s, 59°C for 20 s and 72°C for 20 s). Each real time PCR reaction was done in triplicate.

### Reverse transcription PCR (RT-PCR) analysis

Total RNA was extracted from the lumber spinal cord using Trizol Reagent (Invitrogen, USA). cDNA synthesis was performed by reverse transcription. The primers used were as follows: p300 primer sequences were forward 5′-GCTAATGGAGAAGTGAGGCAGT-3′, reverse 5′-TTTGAGAGGAAGACACACAGGA-3′; COX-2 primer sequences were forward 5′-TGGTGCCGGGTCTGATGATG-3′, reverse 5′-GCAATGCGGTTCTGATACTG-3′. The program for p300 was 94°C for 30 s, 60°C for 30s, 72°C for 30 s and 30 cycles, and it was 94°C for 1 min, 54°C for 1 min, 72°C for 1 min and 30 cycles, for COX-2. β-actin primer sequences were forward 5′-CACGATGGAGGGGCCGGACTCATC-3′, reverse 5′- TAAAGACCTCTATGCCAACACAGT-3′. And the program was 94°C for 30 s, 63°C for 30 s, 72°C for 30 s and 28 cycles. Each PCR sample was incubated for 5 min at 94°C before the initial denaturation and 7 min at 72°C after the last PCR cycle. The products were analyzed by agarose gel electrophoresis containing ethidium bromide. Bands intensity was analyzed and semi-quantified using Quantity One software (Bio-Rad, USA). Results were normalized to β-actin levels.

### Western blot analysis

Proteins were extracted from the spinal cord using the nucleoprotein and cytoplasmic protein extraction kit (Keygen Biotech, China). Each protein sample (20 μg) was separated on an 8% sodium dodecyl sulfate polyacrylamide gel and electrotransferred to a nitrocellulose membrane in Trans-Blot electrophoresis Transfer Cell (Bio-Rad, USA). The membranes were blocked with 5% nonfat dry milk in phosphate-buffered saline with 0.05% Tween 20 for 1 h. Primary antibody incubation (COX-2 dilution: 1:1000, Abcam, USA; p300 dilution: 1:300, Santa Cruz, USA) was performed at room temperature on a shaker for 1 h and then at 4°C overnight, followed by incubation with peroxidase conjugated secondary antibody (1:5000, Sigma, St Louis, MO, USA) for 1 h. Protein brands were visualized by chemiluminescence using an ECL kit (Pierce, USA). Densitometric analysis was carried out using Quantity One software (Bio-Rad), and the results were normalized to loading control.

### Immunocytochemistry analysis

The lumbar spinal cord tissues were post-fixed with 4% paraformaldehyde for 8 h. After dehydration, the tissues were embedded in paraffin and sectioned at a thickness of 5 μm. The sections were dewaxed and treated with 0.01 M citrate buffer at 80°C for 20 min for antigen retrieval and then blocked with 3% goat serum for 1 h. Afterwards, the sections were incubated for 24 h at 4°C with rabbit polyclonal anti-COX-2 antibody (dilution at 1:300; Abcam, USA) or anti-p300 antibody (dilution at 1:300; Santa Cruz, USA), followed by incubation with biotinylated anti-rabbit IgG (dilution at 1:2,000; vector). After being processed with Elite Vectastain ABC kit (Vector), the immunoprecipitates were developed with diaminobenzidine (DAB, Vector). Negative controls lacked the primary antibody. Images of immunocytochemistry were digitally captured using a Nikon N-STORM Professional CCD Camera. Cell counting was performed by two observers unaware of animal treatments. For colocalization, the red dihydroxyfluorane (dilution at 1:100; 16010045) and green dihydroxyfluorane (dilution at 1:200; 16010084) were obtained from Jackson ImmunoResearch Laboratories (West Grove, PA). Double-labeled sections were scanned with a Leica confocal laser scanning microscope (TCS SP5, Mannheim, Germany).

### Statistical analysis

Data were reported as mean ± SEM. CHIP measurements, p300, and COX-2 expression among groups were analyzed with the one-way analysis of variance (ANOVA) followed by Student-Newman-Keuls post hoc test. Behavioral data were assessed by two-way ANOVA with repetitive measurements with group and time post-CCI surgery as variables followed by Holm-Sidak post-hoc analysis. The significance level was set at P < 0.05.

## Abbreviations

ANOVA: Analysis of variance; CBP: CREB-binding protein; CCI: Chronic constriction injury; ChIP: Chromatin immunoprecipitation; CFA: Complete Freund’s adjuvant; COX-2: Cyclooxygenase-2; GABA: Gamma-aminobutyric acid; GFP: Green fluorescent protein; HAT: Histone acetyltransferase; HDAC: Histone deacetylase; LV-NC: Lentiviral vectors encoding small interfering RNA as the negative control; LV-shp300: Lentiviral vectors encoding small interfering RNA specifically against the p300 gene; mGlu2: Metabotropic glutamate 2; MWT: Mechanical withdrawal threshold; NS: Normal saline; p300: E1A binding protein p300; RT-PCR: Reverse transcription-polymerase chain reaction; shRNA: Small hairpin RNA; siRNA: Small interference RNA; TWL: Thermal withdrawal latency.

## Competing interests

The authors report no potential conflicts of interest.

## Authors’ contributions

XYZ participated in experimental design, performed the experiments, analyzed the data and wrote the manuscript. CSH participated in experimental design and performed the experiments. QL performed the experiments. RMC analyzed the data and provided scientific advice. ZBS and WYZ provided scientific advice. QLG conceived of the project and participated in experimental design. All authors approved the final version of the manuscript.
